# Ducts-first stenting strategy for zonal hybrid resection of an ampullary neuroendocrine tumor

**DOI:** 10.1055/a-2860-0670

**Published:** 2026-06-11

**Authors:** Keyang Zhang, Shuqian Hu, Xueting Zhang, Min Min, Yan Liu

**Affiliations:** 1 The School of Medicine 12538 Nankai UniversityTianjinChina; 2 Department of GastroenterologyThe First Medical Center of Chinese PLA General HospitalBeijingChina

Ampullary lesions with bile duct dilatation raise concerns for malignancy or intraductal extension and may lead to surgical referral 1 ; yet, obstruction can also result from papillary occlusion or extrinsic compression and be relieved endoscopically with a minimally invasive, organ-preserving approach 2 . In routine practice, resection-first papillary techniques may cause pancreaticobiliary duct injury and orifice retraction, resulting in loss of duct access and difficult cannulation 3 . Therefore, we performed a ducts-first strategy, involving endoscopic retrograde cannulation with plastic stent placement first, followed by zonal hybrid papillary dissection ( Video 1 ).

Hybrid resection of an ampullary neuroendocrine tumor after pancreaticobiliary cannulation.Video 1

**Fig. 1:**
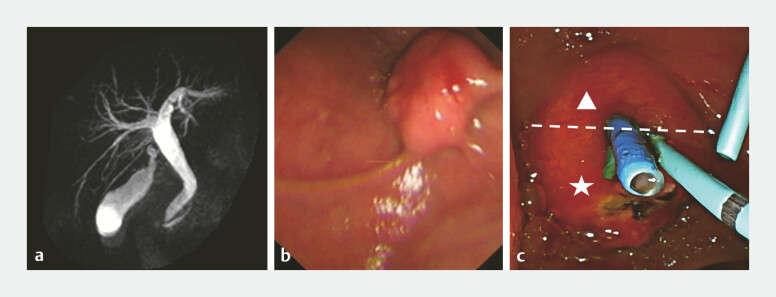
**a** Magnetic resonance cholangiopancreatography. **b** An endoscopic view of the papillary leison. **c** Pancreaticobiliary plastic stents dividing the lesion into suprapapillary (triangle) and infrapapillary (star) components.

**Fig. 2:**
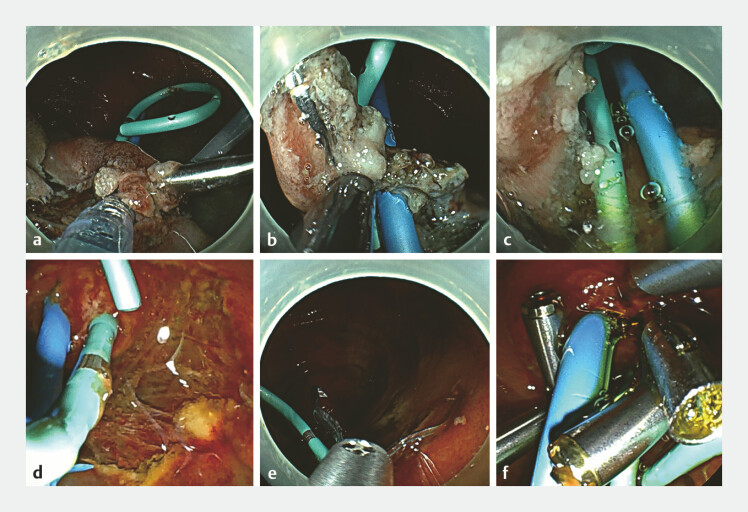
Zonal hybrid resection and defect closure. **a** Endoscopic submucosal dissection of the suprapapillary component assisted using a traction device. **b** and **c** Careful dissection along the biliary and pancreatic stents to preserve duct access. **d** Infrapapillary defect. **e** Defect closure using novel two-teeth clips. **f** Complete closure.

A 51-year-old man was admitted for a tumor of the major duodenal papilla. Magnetic resonance cholangiopancreatography showed mild intra- and extrahepatic bile duct dilatation with low biliary obstruction ( Fig. 1 **a** ). Endoscopic retrograde cholangiopancreatography under general anesthesia was performed first to reveal the papillary tumor ( Fig. 1 **b** ) and relieve the obstruction, followed by the placement of pancreatic and biliary plastic stents to secure duct access and maintain drainage. Three days later, zonal hybrid papillary dissection was performed. The lesion measured about 2.5 cm and was divided by the stents into suprapapillary and infrapapillary components ( Fig. 1 **c** ). The suprapapillary component was resected by endoscopic submucosal dissection using a novel traction device ( Fig. 2 **a** ). Careful dissection was then performed around the biliary and pancreatic stents to preserve both ductal orifices ( Fig. 2 **b** and **c** ). The infrapapillary component was removed with a snare, leaving a clean defect ( Fig. 2 **d** ). Novel high-force clips were deployed to achieve secure closure ( Fig. 2 **e** and **f** ). The patient resumed diet on day 6 and was discharged on day 9 without adverse events. Histology confirmed a neuroendocrine tumor. At 1-month follow-up, the resection site was completely healed and both stents were removed.

In conclusion, this duct-first, zonal hybrid papillary resection secures pancreaticobiliary access and drainage before dissection, facilitating safe resection and closure while potentially reducing technical failure and post-resection complications in selected ampullary lesions.

Endoscopy_UCTN_Code_TTT_1AQ_2AD_3A
